# Managing humidity support in intubated ventilated patients with coronavirus disease 2019 (COVID-19)

**DOI:** 10.1017/ice.2020.418

**Published:** 2020-08-12

**Authors:** Hui-Ling Lin, James B. Fink, Ying-Huang Tsai, Gwo-Hwa Wan

**Affiliations:** 1 Department of Respiratory Therapy, Chang Gung University, Taoyuan, Taiwan; 2 Department of Respiratory Care, Chang Gung University of Science and Technology, Chiayi, Taiwan; 3 Department of Respiratory Therapy, Chiayi Chang Gung Memorial Hospital, Chiayi, Taiwan; 4 Aerogen Pharma, San Mateo, California, United States; 5 Division of Pulmonary and Critical Care Medicine, Department of Medicine, Linkuo Chang Gung Memorial Hospital, Taoyuan, Taiwan; 6 Division of Pulmonary and Critical Care Medicine, Xiamen Chang Gung Hospital, Xiamen, Fujian, China; 7 Department of Obstetrics and Gynaecology, Taipei Chang Gung Memorial Hospital, Taipei, Taiwan; 8 Center for Environmental Sustainability and Human Health, Ming Chi University of Technology, Taishan, New Taipei, Taiwan

*To the Editor*—Humidification is vital in supporting the airways of intubated and mechanically ventilated patients. We address the technical and practical issues of humidification methods selection, with recommendations to minimize bioaerosol dispersion during mechanical ventilation of infected patients with severe coronavirus disease 2019 (COVID-19)–related acute respiratory distress.

Severe pneumonia or sepsis has an estimated 20% occurrence with COVID-19,^1^ and these conditions often require intubation and mechanical ventilatory support. Clinical reviews have recommended the use of a heat and moisture exchanger (HME) for humidification during mechanical ventilation for intubated patients with infectious disease to minimize the risk of exposure the pathogens to healthcare workers (HCWs).^2,3^ HMEs are designed to capture heat and humidity from exhaled gas and to condition inhaled gas to a temperature of 29–32°C with 29–32 mg/L absolute humidity. A systematic review comparing the use of an HME with a heated humidification system during mechanical ventilation showed no difference in artificial airway occlusion, mortality, or pneumonia rate. However, the work of breathing was greater in the HME group, possibly due to increased resistance to gas flow.^4^ Additionally, HME is not recommended for use in patients with copious, thick secretions or low tidal volumes, which have been associated with mechanical ventilation of COVID-19 patients. Patients managed under lung-protective strategies likely have low tidal volume, and the additional dead space of an HME may further increase the ventilation requirement as well as increase the partial pressure of carbon dioxide.^1,2^

Humans produce exhaled breath particles (EBP) ranging from 0.3 to 2.0 μm.^4^ EBP concentrations are positively associated with tidal volume, ventilation ratio (tidal volume to vital capacity), deep exhalation, and breath volume.^4-6^ A Taiwanese study found that the EBP concentration from mechanically ventilated patients were in the range of 0.47–2,554 particles per breath. Most EBPs are <5 μm, and 80% of them range between 0.3 and 1 μm.^7^ Therefore, a bacterial/viral filter should be routinely placed at the end of expiratory limb of the ventilator circuit to prevent the pathogen-containing EBPs from spreading to the hospital environment.

Simple HMEs may allow up to 60% of medical aerosol to pass through,^8^ so only the use of HME with an electrostatic bacterial filter (HMEF) should be considered to reduce exhaled pathogens from intubated patients during mechanical ventilation. There are 2 types of bacterial filters: pleated hydrophobic filters and electrostatic filters.^9^ The efficiency of electrostatic bacterial filters is greatly affected by humidity, in addition to internal volume, resistance, and fully saturated time. When gas pressure is applied to a wet filter in the ventilator circuit, the bacteria and viruses on the filter might be carried through the filter. Furthermore, the condensate in an HMEF might increase gas flow resistance and the risk blockage due to ingress and absorption of the water. A pleated hydrophobic filter is more suitable than an electrostatic filter in a humidified ventilator system.^9^ We strongly recommend using a high-efficiency particulate air (HEPA) filter at the expiratory port of the ventilator as a superior alternative to an HMEF for pathogen filtration in a humidified ventilator system.

One advantage for choosing an HME over heated humidification is the prevention of contaminated condensate spray generated when the ventilator circuit is disconnected from the patient. However, this benefit has not been characterized. During circuit disconnection, sudden depressurization generates shear-force from high ventilator gas flow, resulting in the expulsion of potentially contaminated condensates as bioaerosols. The dual-limb heated ventilator circuits have thermal insulation and breathable properties (ie, Evaqu ventilator circuit) to reduce the condensate production in the circuits. One study showed that both a conventional reusable ventilator circuit system and a disposable ventilator circuit system combined with an autofilled heated humidifier and a closed suction catheter grew a large number of bacteria in the ventilator circuits.^10^ Thus, it is necessary to minimize the need for disconnecting the circuit from the ventilator, which greatly reduces the pathogen exposure via droplets and contact transmission to HCWs.

Intubated, mechanically ventilated COVID-19 patients who develop thick secretions may need to receive heated humidification rather than HME. In clinical practice, single-limb heated-wire ventilator circuit is more commonly used, and the system is sensitive to the surrounding temperature, generating a large volume of condensates in the circuit, especially in the expiratory limb of the ventilator circuit system. We recommend the use of aerosol precautions by HCWs while opening the circuit when changing the HME or ventilator circuit to decrease the risk of exposure to bioaerosols. When changing the HME or dual-limb heated ventilator circuit, the ventilator should be placed on “stand-by” or “off” mode before disconnecting the circuit from the patient. The end of circuit should be capped immediately to prevent the expulsion of bacteria- or virus-containing EBPs from the patient.

In conclusion, to protect HCWs caring for COVID-19 patients during mechanical ventilation, the of using an HME or a dual-limb heated ventilator circuit with minimal condensate production should be considered. The selection should made according to the patient’s minute ventilation and the amount and properties of secretions to provide adequate inhaled gas temperature and humidity. To protect HCWs, a pleated hydrophobic filter with at least 99.97% filtration efficiency should be placed in the expiratory limb of the ventilator, and it should not be replaced by an HMEF in a mechanical ventilation system.
